# One New Genus and Four New Species of Beaded Lacewings (Neuroptera: Berothidae) from Upper Cretaceous Myanmar Amber

**DOI:** 10.3390/insects15040259

**Published:** 2024-04-09

**Authors:** Yuting Chen, Zihao Peng, Siting Liu, Chaofan Shi, Dong Ren, Qiang Yang

**Affiliations:** 1 School of Life Sciences, Key Laboratory of Conservation and Application in Biodiversity of South China, Guangzhou University, Guangzhou Higher Education Mega Center, #230 Waihuanxi Road, Guangzhou 510006, China2112214031@e.gzhu.edu.cn (Z.P.);; 2 School of Earth Sciences and Engineering, Guangdong Provincial Key Lab of Geological Processes and Mineral Resources, Sun Yat-sen University, Guangzhou 510275, China; 3 College of Life Sciences and Academy for Multidisciplinary Studies, Capital Normal University, Xisanhuanbeilu 105, Haidian District, Beijing 100048, China; rendong@mail.cnu.edu.cn

**Keywords:** Berothidae, Myanmar, new genus, swelled flagellomere

## Abstract

**Simple Summary:**

Berothidae, a small family with both extant species and extinct species of Neuroptera, has high species diversity in Cretaceous Myanmar amber. An in-depth study on the diversity of berothids in Cretaceous Myanmar amber will be conducive to better understand the early evolution of Berothidae species and explore the evolutionary relationship of fossil and extant groups. In this study, we describe one new genus and four new species of Berothidae from mid-Cretaceous Myanmar amber. The new genus possesses some synapomorphies in common with Cyrenoberothinae, and this suggests that the new genus may have a close relationship with Cyrenoberothinae.

**Abstract:**

In recent years, as more and more fossil species of berothids from Myanmar have been reported, the species and morphological diversity of Berothidae continues to increase. Herein, one new species of Berothidae, *Aggregataberotha paucipunctata* sp. nov., and one new genus, *Sejunctaberotha* gen. nov., with three new species (*Sejunctaberotha sphaerica* gen. et sp. nov., *Sejunctaberotha tenuis* gen. et sp. nov. and *Sejunctaberotha transversa* gen. et sp. nov.) are described from mid-Cretaceous Myanmar amber. *A. paucipunctata* sp. nov. is assigned to *Aggregataberotha* Wang, Huang & Wang, 2022, based on the characteristics of the similar female terminalia and wing venation, but can be different from *A. punctate* regarding the pale pterostigma and a few detailed features of wing venation. Additionally, representatives of *Sejunctaberotha* gen. nov. are remarkably different from the representatives of the other genera within Berothidae in the configuration of wing venation. For example, *Sejunctaberotha* gen. nov. has simple subcostal veinlets, obviously free Sc and RA at the apex present both in fore- and hindwings, a single ra-rp crossvein connecting the RA with RP3, a single rp-m crossvein locating before the origin of the MP, a simple CuP and no gradate veins. Interestingly, in one of the specimens of *Sejunctaberotha* gen. nov., a pair of spherical bulges was found at the end of the antennae. The new genus *Sejunctaberotha* gen. nov. suggests that Berothidae had a higher potential diversification during the Mesozoic Era.

## 1. Introduction

Beaded lacewings (Neuroptera: Berothidae), belonging to Neuroptera, are generally considered to be a small insect group based on a relatively minor number of extant species. Heretofore, Berothidae (excluding Rhachiberothidae) comprises 118 extant species in 25 genera [1,2], which are distributed in all zoogeographical fauna of the world but are generally distributed in warmer temperate areas, with a few present in subtropical and tropical areas [3,4]. The extant berothids are presently divided into six subfamilies, i.e., Trichomatinae, Berothinae, Protobiellinae, Nyrminae, Cyrenoberothinae and Nosybinae [2]. Rhachiberothidae used to be considered as a subfamily of Mantispidae due to the raptorial forelegs, while some authors take it as a subfamily of Berothidae based on the similar wing venation [5,6,7]. However, research based on the morphology and molecule data suggested that Rhachiberothidae is a separate family, which is a sister group to Berothidae [8]. In this study, we follow Apsöck in regarding Rhachiberothidae as a separate family within Mantispoidea [9].

Compared with the extant genera, the fossil berothid comprises many more genera. Until now, fossil berothids were already reported with 54 species in 34 genera from the Late Triassic to the Upper Eocene [10,11,12,13,14,15]. Among them, 16 berothid genera with 27 fossil species have been described from mid-Cretaceous Myanmar amber [10,12]. The earliest berothids have been reported from the Late Triassic of Japan, where they are represented by the monotypic genus *Triassoberotha* Khramov, Oyama, Kenji & Takahashi, 2023 [13]. Furthermore, Khramov suggested that Mesoberothidae should be considered as a subjective junior synonym of Berothidae, that the genera of Mesoberothidae should be transferred to Berothidae, *Mesoberotha* Riek, 1955, and that *Ferganoberotha* Khramov, 2023, *Triassoberotha* Khramov, Oyama, Kenji & Takahashi, 2023, formed the early group of Berothidae [13,14,15].

Herein, a new genus *Sejunctaberotha* gen. nov., with three species included, and a new species, *Aggregataberotha*, are described from mid-Cretaceous Myanmar amber. The new genus has a close relationship with Cyrenoberothinae, as it shares some similar synapomorphies with Cyrenoberothinae.

## 2. Materials and Methods

This study is based on four specimens from Myanmar amber. The amber pieces were collected from the Hukawng Valley (the state of Kachin in northern Myanmar). The map of the Hukawng Valley was provided by Grimaldi et al. [16]. The volcaniclastic matrix of the amber is estimated to be approximately 98.79 ± 0.62 Ma, i.e., earliest Cenomanian, near the Albian/Cenomanian (Early/Late Cretaceous) boundary [17]. As reported by Cruickshank and Ko, a palynophase analysis of the surrounding rocks of the Myanmar amber layer provided direct biological evidence supporting that Cretaceous amber was formed in the mid-Cretaceous [18]. The biological inclusions of Myanmar amber represent a sample of a tropical forest community in equatorial southeastern Asia at a paleolatitude of approximately 12° N [16,19,20,21,22]. The specimens were permanently deposited in the collections of the Key Laboratory of Insect Evolution and Environmental Changes, College of Life Sciences, Capital Normal University, Beijing, China (CNUB; Dong Ren, Curator).

The specimens were examined under a Zeiss Discovery V20 stereomicroscope (Carl Zeiss, Oberkohen, Germany) and a Nikon SMZ1270 stereomicroscope (Nikon corporation, Tokyo, Japan) and imaged by an AxioCam HRc camera and an iMG SC600C digital camera attached to the Zeiss Discovery V20 (Carl Zeiss, Oberkohen, Germany) and Nikon SMZ1270 stereomicroscope (Nikon corporation, Tokyo, Japan) separately. The figures were processed on Adobe Illustrator CC 2018 and Adobe Photoshop CS6.

Terminology of venation normally follows Kukalová-Peck and Lawrence as interpreted by Yang et al. [23,24,25]. The details of venational terminology follow Oswald [26]. Genital terminology follows Aspöck and Aspöck [27]. Venational abbreviations are as follows: AA1–AA3, first to third branches of the anterior anal vein; CuA, anterior cubitus; CuP, posterior cubitus; MA and MP, anterior and posterior branches of the media; RA, anterior radius; RP, posterior radius; RP1, proximal-most branch of RP; RP2, branch of RP distal RP1; RP3, distal-most branch of RP; Sc, subcosta; ScA, subcosta anterior; and ScP, subcosta posterior.

## 3. Results

### Systematic Palaeontology

Order Neuroptera Linnaeus, 1758.

Family Berothidae Handlirsch, 1906.

Genus *Aggregataberotha* Wang, Huang & Wang, 2022 [10].

Type species. *Aggregataberotha punctate* Wang, Huang & Wang, 2022 [10].

*Aggregataberotha paucipunctata* Chen, Shi, Ren & Yang sp. nov.

urn:lsid:zoobank.org:act:FFB30C0B-33D1-40A1-A7B1-B954C41FB92E

**Figure 1:**
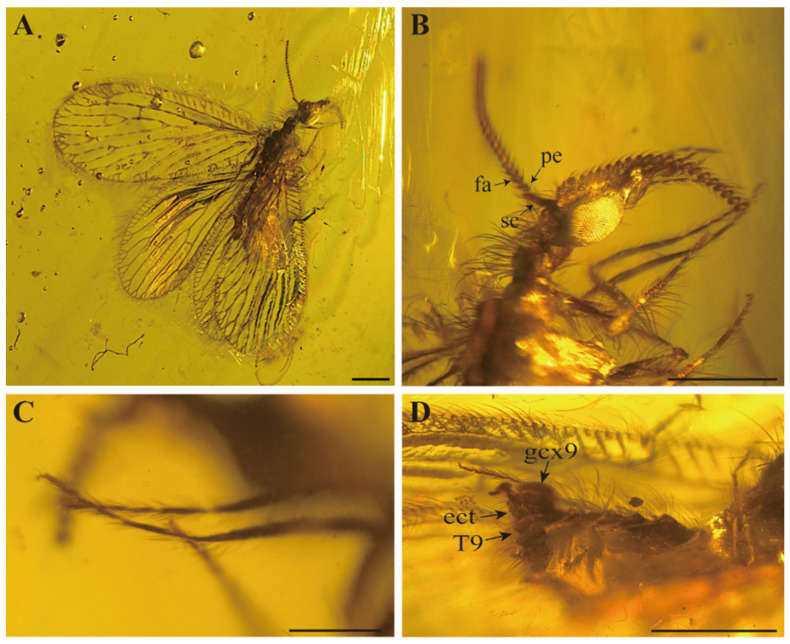
*Aggregataberotha paucipunctata* sp. nov., holotype (CNU-NEU-MA2018096). (**A**) Habitus, lateral view; (**B**) details of antenna, photograph showing scapus (sc), pedicellus (pe) and flagellomeres (fa); (**C**) details of leg; (**D**) details of lateral view of female genitalia, photograph showing gonocoxite 9 (gcx9); free ectoproct (ect) and 9th tergites. Scale bars: (**A**,**C**) 0.2 mm; (**B**,**D**) 0.5 mm.

**Figure 2:**
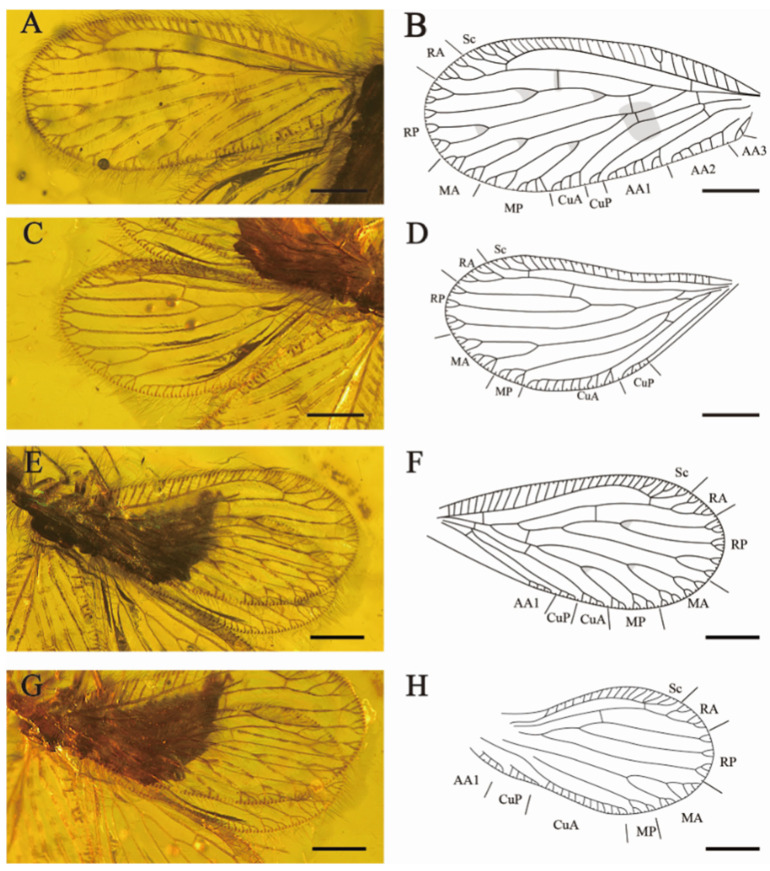
*Aggregataberotha paucipunctata* sp. nov., holotype (CNU-NEU-MA2018096). (**A**) Photograph of left forewing; (**B**) line drawing of left forewing; (**C**) photograph of left hindwing; (**D**) line drawing of left hindwing; (**E**) photograph of right forewing; (**F**) line drawing of right forewing; (**G**) photograph of right hindwing; (**H**) line drawing of right hindwing. Scale bars: (**A**–**H**) 0.5 mm.

Figure 1 and Figure 2.

Material. Holotype: CNU-NEU-MA2018096.

Etymology. The specific name is from the Latin *paucipunctatus* (meaning “spotless”), referring to the new species having pale and fewer wing spots than another species of *Aggregataberotha*.

Locality and horizon. Hukawng Valley, Kachin State, northern Myanmar; Lowermost Cenomanian, Upper Cretaceous.

Diagnosis. Forewing with darker pigmentation along wing margin, subcostal veinlets and longitudinal veins with dark intervals or dots, some small light dark speckles at the divergences of veins. Pterostigma pale. In forewings, the proximal branch of RA forked distally, another branch pectinately branched near wing margin; RP1 with light fork before margin branched; MP with deep fork before margin branched; one rp-m and one m-cua present before origin of MP; one cu-aa1 crossvein present after origin of CuP. In hindwings, one short and sigmoidal rp-m crossvein present, one short and straight mp-cu present.

Description. Body minute size. Body length ca. 3.2 mm. Right forewing length 2.9 mm, width 1.3 mm; left forewing length 2.9 mm, width 1.4 mm. Left hindwing length 2.3 mm, width 1.1 mm; right hindwing length 2.4 mm, width 1.1 mm.

Head (Figure 1B). Medium position of vertex moderately elevated, bearing some mid-length setae interspersed with few short setae. Oculus oval and well-developed, located on both sides of the head and strongly prominent. Frons elongated and with short setae. Palpus and palpi elongated and slender. Antennae with abundant short setae; scapes slender and medium length, about 2.5 times as long as wide; pedicels oblong; flagellum with about 19 articles and posterior part may be broken, flagellomeres bead-shape.

Thorax. Pronotum slender, about 1.2–1.5 times as long as wide, grouped with long setae. Mesonotum well-developed and subsquare shape. Metanotum shorter than mesonotum, subrectangle and dispersedly covered with cluster setae.

Legs (Figure 1C). Femurs and tibias slightly expanded in the middle, covered with middle intermixed with short hairs; mesocoxa elongated, stout and tapered; relative length proportion of protarsomeres 1.8–0.8–0.6–0.5–0.7; relative length proportion of mesotarsomeres 4.1–1.4–0.9–0.8–1.3; tibial spurs absent, two curved and thin tarsal claws.

Female (Figure 1D). Tergite and sternite both closely arranged, tile-like arrangement, moderately sclerotizated, covered with medium-length setae; tergite 7 trapezoid, strong bulge at the end; tergite 8 subrectangle, sternite 8 reduced to small subrectangle; tergite 9 ribbon-shaped, longer than tergite 8, extended to the ventral side; paired 9th gonocoxites, ectoproct obviously separated with tergite 9, subrectangular; hypocaudae absent.

Forewings (Figure 2A,B,E,F). Wing veins diverge with mottled markings. Most subcostal veinlets simple, three subcostal veinlets forked at distal, the rest simple. Sc and RA free distally, one sc-ra and one sc-r crossvein present; one branch of RA forked distally, another branch pectinately branched near wing margin. RP diverged from RA far from wing base, one ra-rp crossvein lies between the separation of RP1 and RP3, RP1 with light fork before margin forks, branches of RP2 and RP3 bifurcated or trifurcated branched. One rp-m crossvein present. Each branch of MA and MP with pectinate margin forks; MP deeply forked before the margin forks (left forewing, LFW). One rp-m present before the origin of MP. Two m-cu crossveins present. CuA with four short and simple pectinate branches near the wing margin, CuP dichotomous forked distally (LFW). One oblique cup-aa1 present. AA1 with five short pectinate branches and one branch forked, AA2 with six pectinate branches, AA3 simple (LFW). MA ending in two main branches twice dichotomously forked; MP deep fork, the proximal branch dichotomously forked, the distal branch pectinately branched; CuA and CuP pectinate branched near wing margin; AA1 simply pectinate branched (right forewing, RFW).

Hindwings (Figure 2C,D,G,H) elongated and round. Two subcostal veinlets forked near the apex of Sc. Sc and R free distally. One ra-rp crossvein present, RP with two bifurcated branches and one trifurcated branch. One short and sigmoidal rp-m crossvein present. MA dichotomous forked, each branch again pectinately branched near wing margin. MP forked before marginal dichotomously branched. One oblique mp-cua crossvein present beyond origin of MP. CuA with nine short, simple pectinate branches and one branch forked (LHW), with twelve simple pectinate branches (RHW), parallel to the posterior of wing margin. CuP with at least four simple branches. Anal vein with four simple and short pectinate branches.

Remark. *Aggregataberotha paucipunctata* sp. nov. belongs to *Aggregataberotha* based on the following characteristics: (1) mottled markings or dots along subcostal veinlets and longitudinal veins, (2) RP with three main branches, (3) most costal crossveins simple and humeral vein not recurrent, (4) one ra-rp present between origin of RP1 and RP3 in forewings but located at RP3 in hindwings, (5) gradate series absent both in fore- and hindwings, (6) stem of M joined with R near the basal one fifth of the wing length, and then divergent from RP, MA and MP both deep dichotomously branched, (7) paired gonocoxite 9 boat-like laterally and without hypocaudae, (8) ectoproct and tergite 9 distinctly unfused in female.

*A. paucipunctata* sp. nov. is obviously distinguished from *A. punctate* by the following characteristics of the venation of wings: in forewing, (1) pale pterostigma present, nearly transparent light black patch at the divergences of veins (vs. dark and distinct pterostigma, fuscous speckles present in *A. punctate*), (2) one cup-aa1 present beyond the origin of CuP (vs. cup-aa1 absent in *A. punctate*); in hindwings, (1) a sigmoidal m-cu located near the wing base, (2) one oblique and short mp-cua present beyond the origin of MP (vs. mp-cua absent in *A. punctate*).

Genus *Sejunctaberotha* Chen, Shi, Ren & Yang gen. nov.

urn:lsid:zoobank.org:act:3BCF5C46-FA17-4CE9-A1E8-ECA0C9697003

Type species: *Sejunctaberotha transversa* Chen, Shi, Ren & Yang gen. et sp. nov.

Species included: *Sejunctaberotha sphaerica* gen. et sp. nov.; *Sejunctaberotha tenuis* gen. et sp. nov.; *Sejunctaberotha transversa* gen. et sp. nov.

Etymology. The generic name is a combination of the Latin *sejuncta* (meaning “separated”) and *Berotha*, a generic name of the family Berothidae, referring to the abnormal vein character of Sc and R free distinctly both in fore- and hindwings of the new genus. Gender feminine.

Diagnosis. Body small to medium size. Antennae moniliform and antennae with the number of total segments less than 40; scapes not larger elongated. Both in forewings and hindwings, pterostigma pale; without recurrent humeral vein; subcostal veinlets all simple; Sc and RA free distally, connected by a crossvein; after the connection point of the crossvein, RA most with more than three simple marginal branches; only one ra-rp crossvein connected RA and RP3; RP with three major branches; without gradate veins. In forewings, each branch of RP with two to three short and simple branches near wing margin; MA originated from R at basal 1/5 of wing length, MA and MP separate at about the half length of the wing; one rp-m crossvein located before the origin of MP; MP slightly forked before margin branched; two m-cua crossveins present; CuA pectinately branched near wing margin, CuP simple; AA1 with less than four simple branches; AA2 with pectinate branches. Pterostigma pale. Female genitalia simple and all sternites without any modification; tergite 9 and ectoproct not fused; callus cerci, hypocauda and pseudohypocauda absent.

Remarks. *Sejunctaberotha* gen. nov. is most related to three extinct genera, i.e., *Sibelliberotha* Azar & Nel, 2013, *Aggregataberotha* and *Haploberotha* Engel & Grimaldi, 2008, but is obviously different from those by the following combined characteristics: (1) subcostal veinlets all simple (*Aggregataberotha* with more than two forked subcostal veinlets near the terminal of Sc); (2) vein of Sc and RA obviously free at apex both in forewings and hindwings (other fossil genera fused distantly both in fore- and hindwings or only free at hindwings); (3) one ra-rp crossvein connected RA and RP3 (one ra-rp crossvein lies between the separation of RP1 and RP3 in *Aggregataberotha* and *Sibelliberotha*); (4) each branch of RP with two or three short and simple branches near wing margin (*Aggregataberotha* and *Haploberotha* RP with fork before margin branched, *Sibelliberotha* RP only with two short branches near wing margin); (5) in forewings, MP with light fork before margin branched (*Aggregataberotha* MP with deep fork before margin branched, *Sibelliberotha* MP with pectinate branches near wing margin); (6) one rp-m present before the origin of MP (one rp-m present after the orgin of MP and connect RP with MP in *Sibelliberotha* and *Haploberotha*); (6) in forewings, CuP simple (*Sibelliberotha* and *Aggregataberotha* with two to three short branches near wing margin, *Haploberotha* with two forked branches) [10,28,29,30].

*Sejunctaberotha sphaerica* Chen, Shi, Ren & Yang gen. et sp. nov.

urn:lsid:zoobank.org:act:BE7AAEC9-B910-4169-94FD-0E1BF710E556

**Figure 3:**
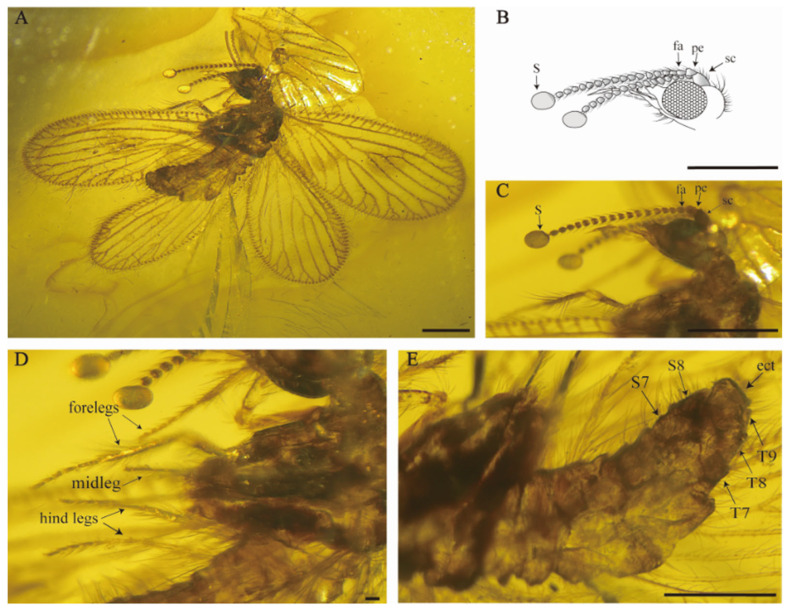
*Sejunctaberotha sphaerica* gen. et sp. nov., holotype (CNU-NEU-MA2018097). (**A**) Habitus, lateral view; (**B**) line drawing of antenna; (**C**) photograph showing details of antenna, photograph showing scapus (sc), pedicellus (pe), flagellomeres (fa) and spherical bulges (S); (**D**) photograph showing forelegs, midleg and hind legs; (**E**) details of the left lateral view of female genitalia, photograph showing free ectoproct (ect) and 9th tergite; S7–S8, 7th–8th sternites; T7–T9, 7th–9th tergites. Scale bars: (**A**–**C**,**E**) 0.5 mm, (**D**) 0.05 mm.

**Figure 4:**
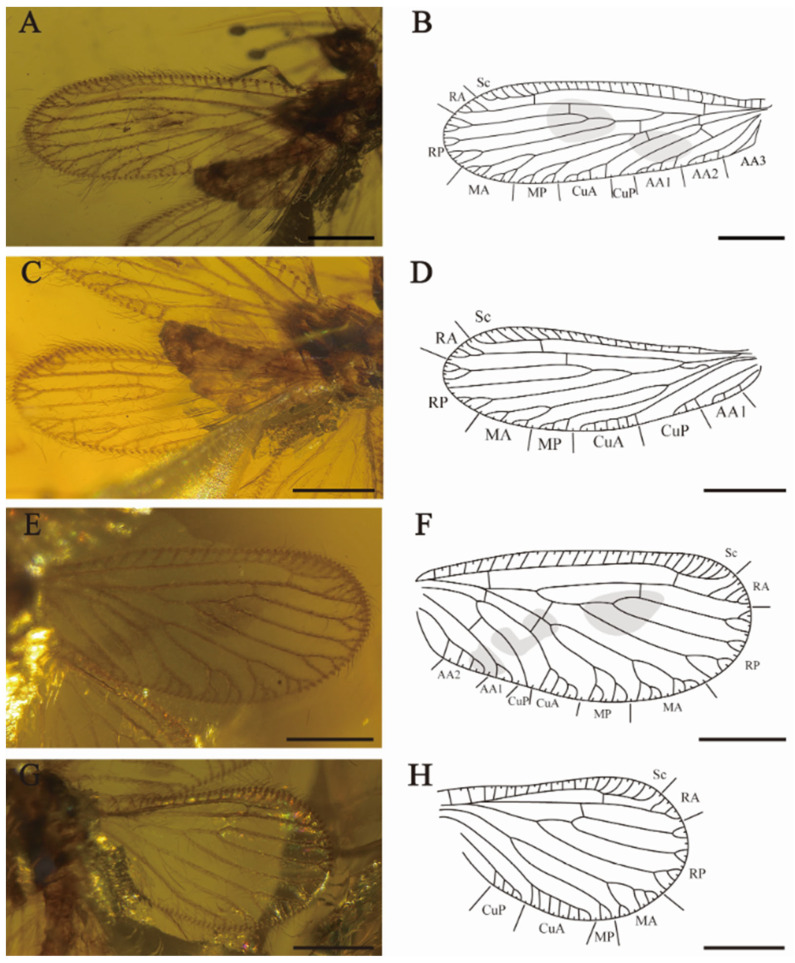
*Sejunctaberotha sphaerica* gen. et sp. nov., holotype (CNU-NEU-MA2018097). (**A**) Photograph of left forewing; (**B**) line drawing of left forewing; (**C**) photograph of left hindwing; (**D**) line drawing of left hindwing; (**E**) photograph of right forewing; (**F**) line drawing of right forewing; (**G**) photograph of right hindwing; (**H**) line drawing of right hindwing. Scale bars: (**A**–**H**) 0.5 mm.

Figure 3 and Figure 4.

Material. Holotype: CNU-NEU-MA2018097.

Etymology. The specific epithet is from the Latin *sphaerica* (meaning “spherical”), referring to a pair of spherical bulges at the ends of the antennae.

Locality and horizon. Hukawng Valley, Kachin State, northern Myanmar; Lowermost Cenomanian, Upper Cretaceous.

Diagnosis. Vertex covered with scattered, short and fine setae. RA with three simple branches at distal; MA dichotomously branched at distal; elongated and sigmoidal crossvein linked RP and M in hindwings; ectoproct very broad and obviously bigger than tergite 9.

Description. Body length ca. 2.2 mm. Right forewing length 2.1 mm, width 0.9 mm; left forewing length 2.5 mm, width 0.7 mm. Left hindwing length 1.9 mm, width 0.7 mm; right hindwing length 1.6 mm, width 0.9 mm.

Head (Figure 3B,C). Vertex moderately swelled, higher than oculus, with abundant long, fine setae; gena expanded; frons and mouthparts elongated, with medium interspersed with small setae. Compound eyes protruded in a hemispherical shape and well developed, antennae moniliform, shorter than body length, bearing ring of short and bent setae; scapus short and stout, about 1.5 times as long as wide; pedicel subsquare, length almost equal to width; flagellum with at least 14 articles, flagellomeres subquadrangular, a pair of spherical bulges present at the end of the antennae.

Body medium size. Pronotum subrectangle, one transverse furrow present, about 1.5 times as wide as long, medium-sized brown setae spread on the later edge. Mesonotum and metanotum with similar shape and color, slightly wider than long.

Legs (Figure 3D) slender; femur slender, short setae covered both at ends while middle part located with mix of stout long and medium-length fine setae; tarsus densely distributed with short setae, with tarsus 5 segmented, the first tarsomere longest; without tibial spurs; tarsal claws slender and curved; arolium present. Forelegs are walking legs. Procoxa elongated, subcylindrical, entire surface covered with short setae, relative length of tarsomeres 2.7–1.7–1.3–1.1–1.9.

Female genitalia (Figure 3E). Abdomen slightly swollen. Segments 1–8 well developed and subrectangle, sternite 7 in lateral view subquadrate, sternite 7 and sternite 8 both one piece, without modification; tergite 9 not elongated, subtriangular and distinctly smaller and shorter than tergite 8; ectoproct one piece, obviously bigger than tergite 9; tergite 9 is distinctly separate from ectoproct, without hypocaudae; tergite and ectoproct bearing scattered, mid-length fine hairs.

Forewings (Figure 4A,B,E,F) elongated oblong with rounded apex, membrane hyaline. Dense medium-sized setae and prominent trichosors along the whole wing margin, fine setae scatteredly located on venation. Costal space narrow at base, first slightly expanded proximate the base of the wing at one quarter of the wing length, maximally expanded in the end of subcostal vein. Recurrent humeral vein absent. All subcostal veinlets simple. Sc and RA free at apex and connected with a crossvein. Two crossveins present between Sc and RA. RA with simple pectinate branches near wing margin. RP diverged from RA far apart from wing base, with three major branches, and one crossvein between RA and RP connecting RA and RP3, RP branches bifurcately or trifurcately branched at apex. MA amalgamated with stem of racial vein near 1 sc-r, one rp-m crossvein present before origin of MP, MA and MP both shallowly forked, with simple distal dichotomies. Two m-cua crossveins present beyond origin of CuP, CuA with four pectinate branches close to posterior wing margin, CuP simple. One cup-aa1 crossvein present near origin of the CuP, AA1 dichotomous branched near wing edge, AA2 with four pectinate branches, AA3 not detected (RFW); AA1 and AA2 with three to four short and pectinate branches, AA3 simple (LFW).

Hindwings (Figure 4C,D,G,H) elongated oval, obviously smaller and shorter than forewings. Costal space narrow at wing base, then typically expanded in pterostigmal region. Subcostal veinlets simple without fork. Sc and RA not fused distally, connected by one sc-r crossvein. RA with three distal simple branches, one ra-rp crossvein connected the RA and RP3, RP with three distal forked branches. One short and sigmoidal crossvein linked RP and M; MA dichotomously branched at distal, each branch bifurcated (LHW), the proximal branch trichotomously forked and the distal branch twice forked (RHW); MP with three simple distal branches (LHW), MP bifurcated (RHW); CuA with six to seven pectinate branches almost parallel to posterior wing edge, CuP with at least four pectinate branches. AA1 with three pectinate branches near wing margin.

*Sejunctaberotha tenuis* Chen, Shi, Ren & Yang gen. et sp. nov.

urn:lsid:zoobank.org:act:7B54281A-9155-4FB2-805C-82F5616B85C3

**Figure 5:**
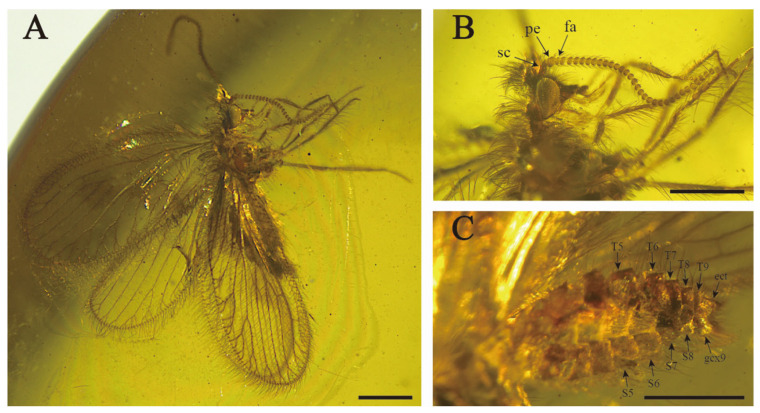
*Sejunctaberotha tenuis* gen. et sp. nov., holotype (CNU-NEU-MA2018098). (**A**) Habitus, lateral view; (**B**) details of antenna, photograph showing scapus (sc), pedicellus (pe) and flagellomeres (fa); (**C**) details of lateral view of female genitalia, photograph showing gonocoxite 9 (gcx9); free ectoproct (ect) and 9th tergite; S5–S8, 5th–8th sternites; T5–T8, 5th–8th tergites. Scale bars: (**A**–**C**) 0.5 mm.

**Figure 6:**
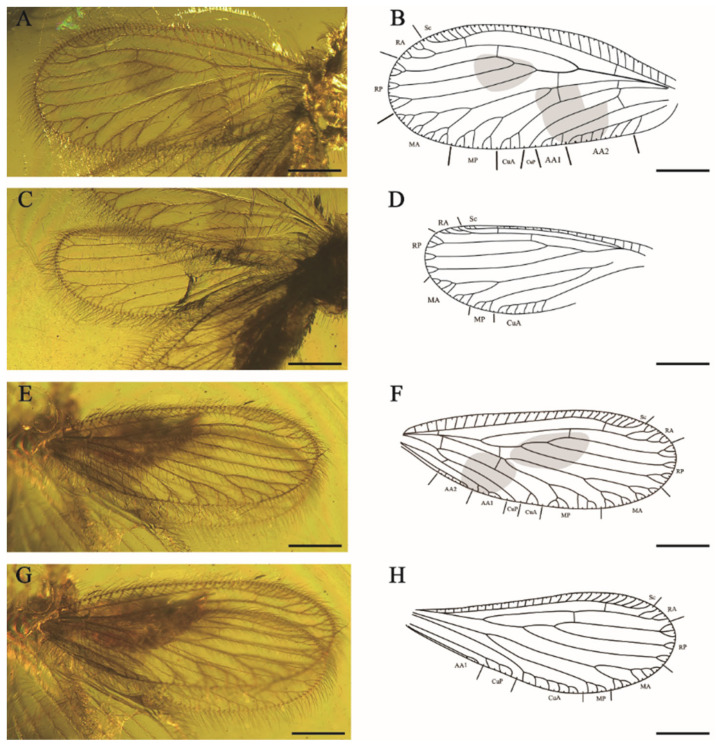
*Sejunctaberotha tenuis* gen. et sp. nov., holotype (CNU-NEU-MA2018098). (**A**) Photograph of left forewing; (**B**) line drawing of left forewing; (**C**) photograph of left hindwing; (**D**) line drawing of left hindwing; (**E**) photograph of right forewing; (**F**) line drawing of right forewing; (**G**) photograph of right hindwing; (**H**) line drawing of right hindwing. Scale bars: (**A**–**H**) for 0.5 mm.

Figure 5 and Figure 6.

Material. Holotype: CNU-NEU-MA2018098.

Etymology. The specific epithet is from the Latin *tenuis* (meaning “thin”), referring to the dorsal plate of the prothorax being narrower and slightly wider than the length.

Locality and horizon. Hukawng. Valley, Kachin State, northern Myanmar; Lowermost Cenomanian, Upper Cretaceous.

Diagnosis. Protothorax slender, longer than wide, with middle length bristle; ectoproct very broad and obviously bigger than tergite 9 and 9th gonocoxites. Vertex and pronotum bearing with clustered long hairs. RA and MA both dichotomously branched in forewings. No sigmoidal or straight crossvein between basal of RP and M in hindwings. Entire wing margin with dense and medium-length hairs.

Description. Body length ca. 1.2 mm. Right forewing length 2.8 mm, width 1.0 mm; left forewing length 2.7 mm, width 1.2 mm. Left hindwing length 2.3 mm, width 1.0 mm; right hindwing length 2.6 mm, width 1.0 mm.

Head (Figure 5A,B). Oculus oval shaped. Vertex slightly prominent, bearing dense, long setae. Frons and mouthparts elongated, shorter than the other species of this new genus. Antenna with short scape, ca. 1.5 times as long as wide, covered with short setae; pedicel subsquare, about 3 times big as the first flagellomere; the first flagellomere flattened shape, the rest of flagellomere globular shape, with 36 segments of flagellomere, the last flagellomere subtriangle.

Body medium size. Pronotum short and slender, bearing dense, long and curved setae. Mesonotum and metanotum both transverse strip shape, notum slightly broader than the metanotum, covered with intensive long setae.

Femur and tarsus slender, covered with short hairs at both ends while long, mixed short hairs located in the middle part; tarsus with five segments covered with short setae; tibial spurs absent; tarsal claws slender and curved, simple; arolium present. First tarsomere of the foreleg 1.5 times as long as second tarsomere, other tarsus of midleg is 2.5 times as long as second tarsus; metafemur subcylindrical, middle part of tarsus slightly swelled.

Female genitalia simple (Figure 5C). Tergite and sternite 1–8 both well developed and subrectangle, without modification; sternite gradually decreases from sternite 6, sternite 8 smallest and reduced to a small subtriangle; ventral side of tergite 9 bent caudally and slightly extended, thinner than tergite 8; ectoproct and 9th gonocoxites both semicircle; tergite 9 free from ectoproct, without hypocaudae.

Forewings (Figure 6A,B,E,F) oval and rounded apex. All subcostal veinlets simple. RA forked and all branches marginally branched. Sc and R free apically, one sc-r and one sc-ra crossvein present. One ra-rp crossvein connecting RA and RP3, RP1 and RP3 simple pectinate branches and RP2 bifurcately branched at the end. One rp-m and m-cua crossvein present before origin of MP. MA and MP both lightly fork with trifurcate branch near wing margin. Two m-cua crossveins present. CuA with three short pectinate branches, CuP simple. One cup-aa1 crossvein present. AA1 with three pectinate branches; AA2 with five to seven simple pectinate branches.

Hindwings (Figure 6C,D,G,H) elongated and round. All subcostal veinlets simple. Sc and R free at apex, connected with a crossvein. One ra-rp crossvein present; RA simple trifurcately branched at apex; RP1 and RP2 bifurcately branched; RP3 trifurcately branched. MA shallow branched with some pectinate branches near wing margin; MP trifurcately branched. CuA with six short pectinate branches near the wing edge, CuP with three or five simple pectinate branches. AA1 with a distal fork.

*Sejunctaberotha transversa* Chen, Shi, Ren & Yang gen. et sp. nov.

urn:lsid:zoobank.org:act:B9493266-1509-4FED-9DDF-549A596848D8

**Figure 7:**
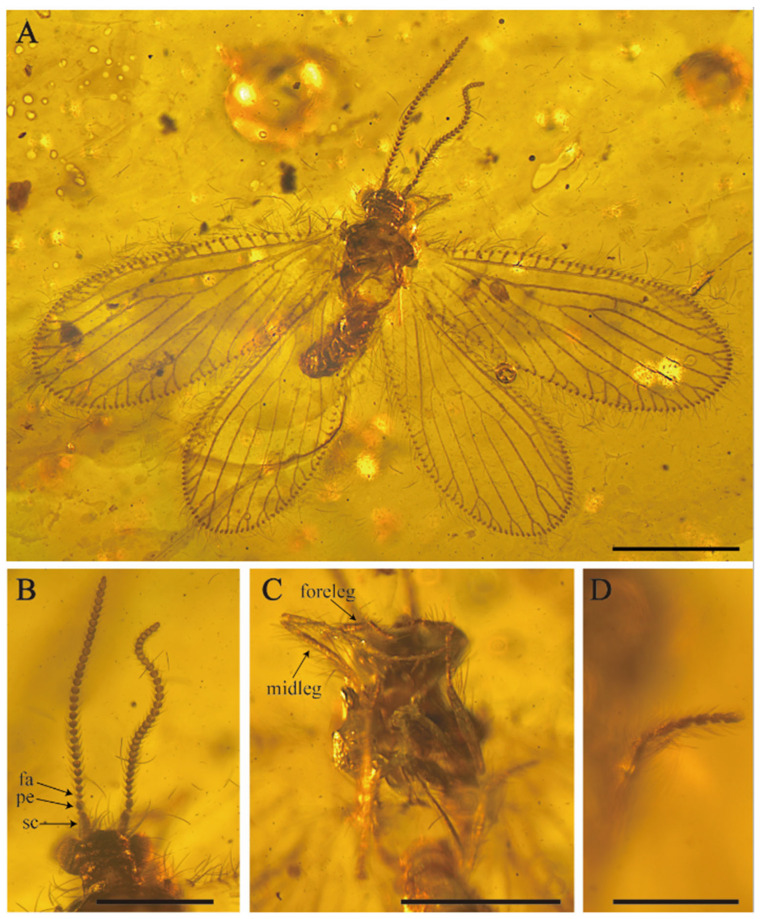
*Sejunctaberotha transversa* gen. et sp. nov., holotype (CNU-NEU-MA2018100). (**A**) Habitus, dorsal view; (**B**) photograph showing the details of antenna, photograph showing the scapus (sc), pedicellus (pe) and flagellomeres (fa); (**C**) photograph showing the details of foreleg and midleg; (**D**) details of the tarsus of hind leg. Scale bars: (**A**–**C**) 0.5 mm; (**D**) 0.2 mm.

**Figure 8:**
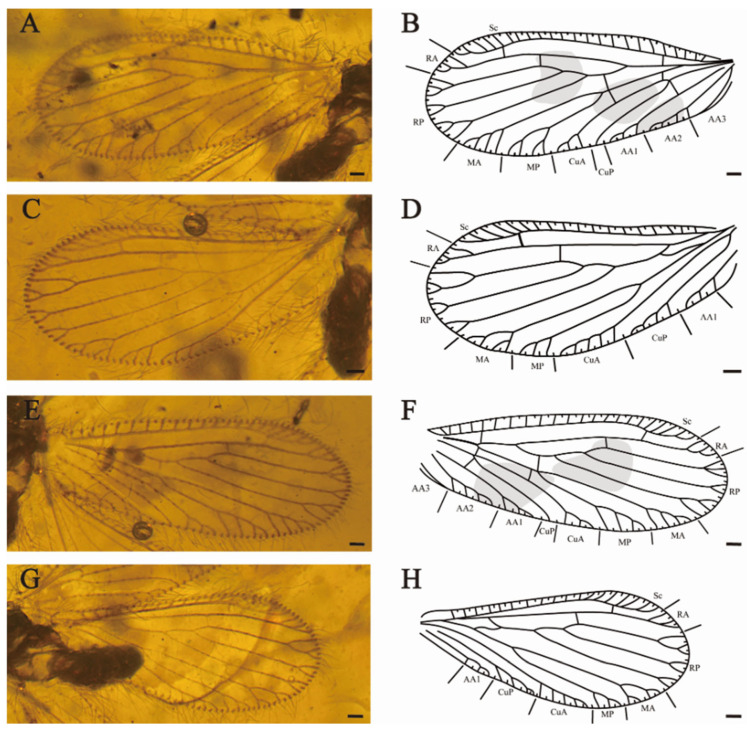
*Sejunctaberotha transversa* gen. et sp. nov., holotype (CNU-NEU-MA2018100). (**A**) Photograph of left forewing; (**B**) line drawing of left forewing; (**C**) photograph of left hindwing; (**D**) line drawing of left hindwing; (**E**) photograph of right forewing; (**F**) line drawing of right forewing; (**G**) photograph of right hindwing; (**H**) line drawing of right hindwing. Scale bars: (**A**–**H**) 0.1 mm.

Figure 7 and Figure 8.

Material. Holotype: CNU-NEU-MA2018100.

Etymology. The specific epithet is from the Latin *transversus* (meaning “transverse”), referring to the dorsal plate of the prothorax stout, transverse rectangle, clearly wider than long.

Locality and horizon. Hukawng Valley, Kachin State, northern Myanmar; Lowermost Cenomanian, Upper Cretaceous.

Diagnosis. Antennae relatively long, with about 29 flagellomeres; protothorax short and stout, wider than length; in forewing, MA pectinately branched near the wing margin in forewing while other species are bifurcately forked and pectinately branched at apex. Protergum stout and transverse, wider than long.

Description. Body length ca. 1.6 mm. Right forewing length 2.3 mm, width 0.8 mm; left forewing length 2.2 mm, width 0.9 mm. Left hindwing length 1.7 mm, width 0.9 mm; right hindwing length 1.9 mm, width 0.8 mm.

Head (Figure 7B). Oculus strongly prominent in hemispherical shape. Frons and mouthparts slightly elongated. Vertex elevated above the oculus, hemisphere, scattered mid-length bristles. Antennae surround with dense bristle; scape slightly elongated, about 2.5 times as long as wide; pedicel subrectangle; flagellomere subtrapezoidal, with ca. 29 flagellomeres, last flagellomere triangle, smaller than previous flagellomeres.

Pronotum transversal square, covered with scattered medium-length setae, one transversal furrow present. Mesothorax and metathorax both damaged.

Forelegs are walking legs. Femur and tarsus slender, short setae covered both at ends while middle part located with mixed of stout, long and medium-length, fine setae; short setae densely distributed in tarsomere, with tarsus 5 segmented, the first tarsomere longest, the remaining tarsal segments nearly equal in length; without tibial spurs; tarsal claws slender and curved; arolium present (Figure 7C,D).

Abdomen poorly preserved.

Forewings (Figure 8A,B,E,F) ovoid and rounded apex. Humeral vein simple. Sc and R free distally, connected by a crossvein, another sc-r crossvein located before origin of RP. One ra-rp3 present. RP1 and RP3 with three short pectinate branches, RP2 one bifurcated, forked branch. One rp-m crossvein present before the origin of MP; MA pectinately branched near wing margin; MP light bifurcated fork before margin branch (LFW), or with four pectinate branches (RFW). Two m-cua crossveins present; CuA trichotomous branched distally; CuP simple. One cu-aa1 crossvein present near the origin of CuP, AA1 and AA2 with three to four short pectinate branches near wing margin; AA3 simple.

Hindwings (Figure 8C,D,G,H). Costal space narrow at wing base, maximum width and rounded at apex. All subcostal veinlets simple. Sc and R broadly separated, connected by one crossvein. RP diverged from RA far from wing base; RP with three major branches, each branch forked at apex. One short and sigmoidal rp-m crossvein present before origin of R1 and MP. MA twice forked distally; MP pectinately branched apically. CuA almost parallel to the posterior wing margin, with five pectinate branches; CuP pectinately branched near the wing margin. AA1 with three short pectinate branches close to the wing verge, AA2 and AA3 not observed.

## 4. Discussion

In the genus *Aggregataberotha*, only one species, *A. punctate*, was described before. According to the original description, the Sc and RA were fused at the apex in the forewings of *Aggregataberotha* [10]. However, it can be clearly observed that the Sc and RA of *A. paucipunctata* sp. nov. are not fused in the forewings, although the terminal of the Sc has a slight downward bending in the pterostigma area. The Sc and RA are connected by a stout crossvein at the apex, and the RA is not significantly thickened behind the crossvein. Meanwhile, the crossvein between the terminal of the Sc and RA is straight in *A. paucipunctata* sp. nov. as the Sc and RA were free at the apex, while this part of *A. punctate* is not well developed. Therefore, we suppose that the Sc and RA are also free at apex in the forewings.

Compared with the extant berothids, most of the genera have one series of gradate crossveins or reticulate crossveins, except *Nosybus* and *Speleoberotha* possess fewer crossveins [1,31], which is similar to the new genus *Sejunctaberotha* gen. nov., also has no gradate crossveins in forewings. However, *Sejunctaberotha* gen. nov. is distinguished from those two extant genera by the simple costal veinlets, which in extant genera are usually forked in forewings.

In both the fore- and hindwings, Sc and RA are mostly fused at the apex among the extinct genera of Berothidae, while distally free of Sc and RA are only present in *Aggregataberotha* and *Sejunctaberotha* gen. nov [10]. Moreover, the characteristic that Sc and RA do not fusing distally in hindwings is present in four Myanmar genera: *Sejunctaberotha* gen. nov., *Protoberotha*, Khramov, 2021, and *Aggregataberotha* [10,32]. However, *Protoberotha*, *Osmyloberotha* and *Aggregataberotha* show distinct differences from *Sejunctaberotha* gen. nov., as follows: *Protoberotha* has only a simple branch in RP and no ra-rp crossvein in fore- and hindwings [32]; *Sejunctaberotha* gen. nov. RP has three major branches in RP, and ra-rp present in fore- and hindwings; *Osmyloberotha* has an elongated wing shape and two series of crossveins present in hindwings; *Sejunctaberotha* gen. nov. has an oval wing shape and no gradate veins [33].

In addition, except for *Sejunctaberotha* gen. nov. and *Aggregataberotha*, distally free Sc and RA in forewings are also present in *Microberotha* Archibald & Makarkin, 2004, and *Pseudosisyra* Makarkin, 1999 [34,35]. The condition of the ends of the Sc and R in *Microberotha* cannot be observed clearly due to its poor preservation [34]; the holotype of *Pseudosisyra* only preserved the forewings with the following combined characteristics: well-developed recurrent humeral vein; mostly forked costal crossveins; branched CuP before marginal twigging; and one crossvein in the anal area [35]. These characteristics are clearly distinguished from *Sejunctaberotha* gen. nov.

In the new genus *Sejunctaberotha* gen. nov., the plesiomorphy, such as separated 9th tergite and ectoproct, simple 9th gonocoxite, elongated frons and mouthparts, suggest that *Sejunctaberotha* gen. nov. is closely related to Cyrenoberothinae. It is still necessary to further study and clarify the relationship between the new genus and Cyreoberothinae through a phylogenetic analysis.

### Special Spherical Bulges at the Tip of Antenna

Antennae are the main mediating organs, with different types of receptors that help insects receive various external signals [36]. They are also closely related to the behaviors of insects, such as habitat search, intraspecific communication and mate searching [37,38]. In insects, swelled antenna ends are usually observed in Lepidoptera and most of them are clavate antennae, and the expansion of the antennae only at the last flagellomere are rare. The antennae of Neurpotera are mostly filiform, moniliform and pectiniform. Among the Mantispoidea, the antennal types are mostly filiform and moniliform, but the antennal type of Ascalaphidae and some Myrmeleontidae species in Myrmeleontoidae is clavate, and gradually swelled flagella are observed near the end of the 11th–13th segments of them.

In this study, an interesting phenomenon is found that a pair of two bulbous protrusions at the end of antennae clearly present in *Sejunctaberotha sphaerica* gen. et sp. nov., which is the first time been observed in berothids. We suppose that there may be two possibilities for this phenomenon: (1) they are specialized structures at the tip of the antennae, but they are without the typical characteristics of other flagellomeres; (2) they are hemolymph droplets that flowed out of the tip of the antennae due to damage or breakage. There was one report about hemolymph droplets from a damaged spider limb in amber [39], but unfortunately, the bubbles also had morphological differences. Therefore, more evidence is still needed to explain it.

## 5. Conclusions

A new genus, *Sejunctaberotha* gen. nov., and four new species, *Sejunctaberotha sphaerica* gen. et sp. nov., *Sejunctaberotha tenuis* gen. et sp. nov., *Sejunctaberotha transversa* gen. et sp. nov. and *Aggregataberotha paucipunctata* sp. nov., are described from mid-Cretaceous (lowest Cenomanian) Myanmar amber. The new genus has representative characteristics that indicate it may have close relationship with Cyrenoberothinae. Furthermore, the new species *Sejunctaberotha sphaerica* gen. et sp. nov., with a pair of spherical bulges at the end of the antennae, has never been found in Berothidae before. This discovery highlights the palaeodiversity of beaded lacewings from the Cretaceous, and it could provide more data for studying the morphological evolution and phylogeny of berothids.

## Data Availability

This published work and the nomenclatural acts it contains have been registered in ZooBank, the online registration system for the ICZN (International Code of Zoological Nomenclature). The LSID (Life Science Identifier) for this publication is urn:lsid:zoobank.org:pub:3C7A81DD-E7EF-4E3E-9663-144560F403E3.
